# Digital finance development and manufacturing emission reduction: An empirical evidence from China

**DOI:** 10.3389/fpubh.2022.973644

**Published:** 2022-08-12

**Authors:** Yikun Yin

**Affiliations:** College of Biological and Agricultural Engineering, Jilin University, Changchun, China

**Keywords:** digital finance, carbon emission, manufacturing, inverted U-shape, scale effect, technological effect

## Abstract

The implementation of carbon peak and carbon neutral goals cannot be achieved without the effective support of digital finance. This paper studies the inverted N-curve relationship between digital finance and carbon intensity, and identifies the emission reduction effects of digital finance development from the enterprise level. The study found that the development of digital finance has an inverted-N dynamic impact on the carbon intensity of the manufacturing industry. The emission reduction effects of the development of digital finance have typical multi-dimensional heterogeneous characteristics at the regional and enterprise levels. Mechanism analysis shows that the emission reduction effect of digital finance development depends on the combined effect of scale and technology. The initial stage of digital finance development can promote carbon emission reduction through the “scale effect” of reduction, and the digital finance development in the intermediate stage can increase carbon intensity through the “scale effect” of expansion, while long-term emission reductions can be achieved based on technological effect caused by the digital finance development in the mature stage.

## Introduction

China announced at the general debate of the 75th session of the UN General Assembly that “China will increase its independent national contribution, adopt more vigorous policies and measures, strive to peak CO_2_ emissions by 2030, and strive to achieve carbon neutrality by 2060.” The choice of the manufacturing sector as the breakthrough point is crucial to the effective implementation of the “dual carbon” targets of peak and neutral carbon emissions. In fact, a single end-of-pipe management model is no longer sufficient to meet the needs of the manufacturing sector to promote energy saving and green growth, and digital finance, which is supported by digital technology and integrated with traditional financial industries with more precise matching efficiency, undoubtedly provides a “new answer” for the manufacturing sector to reduce emissions.

Digital finance is defined as the integration of traditional financial business with Internet technology (1), covering such businesses as electronic payment and online investment and financing. Since the introduction of the policy of “encouraging the development of Internet finance” in 2014, China's digital finance has entered the “fast lane” of development, with mobile payment tools such as WeChat and Alipay developing rapidly and growing. The Digital Inclusive Finance Index measured by the Digital Finance Research Center of Peking University shows that in 2019, China's Digital Inclusive Finance Index exceeded that of 2011 by nearly eight times. Although digital finance has impacted traditional financial services to a certain extent, it is able to have many positive impacts on the development of the real economy, such as the manufacturing industry, based on the low threshold nature of financing, the universality of financial services, the accessibility of service scope and the convenience of mobile payments (2, 3). Zhang et al. (4) found that digital financial development can promote inclusive economic growth by stimulating innovation and entrepreneurship, and the economic growth-enhancing effect of inclusive finance is more significant in rural areas. Yu et al. (5) focused on the impact of digital finance on less developed regions, and the results showed that digital finance can help improve the function of financial services to narrow the development gap between urban and rural areas, thus promoting high-quality economic development. Based on the intrinsic connection between digital finance and manufacturing development, it can be inferred that digital finance will inevitably have a profound impact on carbon emissions in manufacturing production activities (6), so it is of great practical significance to build a low-carbon development model for manufacturing supported by digital finance. Based on this, this paper attempts to integrate digital finance and carbon emissions into a unified analytical framework, assess the emission reduction effects of digital finance development at the manufacturing enterprise level, and examine the intrinsic mechanisms.

The possible marginal innovations of this paper are mainly reflected in the following aspects: (1) This paper systematically examines the non-linear association between digital finance and carbon emission reduction in the manufacturing industry from both theoretical analysis and empirical tests. To overcome the potential endogeneity problem, this paper selects natural geographic and historical data such as the spherical distance from each city to Hangzhou and the number of post offices and fixed telephone ownership in 1984 as instrumental variables for endogeneity analysis. (2) The paper analyzes the intrinsic mechanism of digital finance's impact on manufacturing emission reduction through the “scale effect” and “technology effect,” and explains it based on the alleviation of financing constraints and digital transformation caused by the development of digital finance. The paper also explains the impact of digital finance on manufacturing emissions reduction through the “scale effect” and “technology effect.” At the same time, the dynamic changes in the effect of digital finance on manufacturing emission reduction at different stages of development are examined. (3) This paper examines the heterogeneous impact of digital finance on manufacturing carbon emission reduction from the perspective of regional distribution, resource endowment, enterprise size, enterprise ownership and enterprise age.

## Literature review

### Literature review on the impact of environmental development on financial pollution

As an important support for economic growth, the impact of financial development on environmental pollution has also received much academic attention. He et al. (7) empirically examined the impact of financial development on environmental pollution using technological innovation as a mediating variable, and the results showed that both the scale of credit of financial institutions and the scale of financing in financial markets could significantly affect environmental pollution. Although the correlation between financial development and environmental pollution has been verified, it is still controversial whether the impact of financial development on environmental pollution is improved or worsened.

Tamazia et al. (8) focused on the financial markets of BRICS countries and found that the reduction of financing costs and the expansion of financing channels provided sufficient financial support for technological research and development, thus significantly reducing carbon emissions in BRICS countries. Hassan and Asall (9) found that financial development can significantly reduce the rate of environmental degradation. Using South Africa as an example, Shahbaz et al. (10) and Salahuddin et al. (11) found that financial development in developing countries can lead to a greater flow of funds to environmentally friendly projects and ultimately reduce pollution. Xu et al. (6) reported that digital finance has the effect of pollution reduction, and the entrepreneurship effect, innovation effect and industrial upgrading effect are important mechanisms. Zhang and Chen (12) found that financial development can coordinate with environmental regulation to promote green economic transformation under the circumstance that environmental regulation fails to play a significant role in policy. Zheng et al. (13) suggested that the digital finance has significant governance effect on environmental pollution. The digital finance can indirectly control environmental pollution by promoting social and economic development, leading industrial transformation and upgrading, and improving the level of green technology innovation.

However, some scholars pointed out that the digital finance can lead to increased pollution.

Sadorsky (14) argued that financial development increases the financing capacity of enterprises, which in turn led to the expansion of production capacity and energy consumption, ultimately leading to increased environmental pollution. Similarly, Tao et al. (15) compared the pollution control effects of financial scale and financial efficiency, and found that the increase in financial efficiency can create incentives for clean product development and hence emission reduction. Sehrawat et al. (16) pointed out that the expansion of consumption in the middle-income group and the resulting increase in carbon emissions are particularly evident in the context of financial development. Zou (17) further suggested that green-oriented financial policies also promote the emission reduction behaviors of enterprises. Song and Bian (18) argued that the direct effect, indirect effect and total effect of financial opening on haze pollution are significantly positive in the short term, that is, the higher the degree of financial openness, the higher the haze pollution level.

Given the uncertain relationship between financial development and environmental pollution, it has become a more common choice to study the intrinsic association between the two from a non-linear perspective. Financial development can promote technological progress and enhance technical support for carbon emission reduction, but it will also lead to an increase in economic growth rate and energy consumption, resulting in an increase in total carbon emissions. Hu and Yi (19) decomposed the environmental effect of financial development into scale effect and technology effect. Ren and Zhu (20) further took into account the structural effect of financial development on environmental pollution, and the results showed that there were differences in the effects of financial development on different pollutants. Wang et al. (21) further distinguished the non-linear relationship between financial scale, financial efficiency and environmental pollution, in which financial scale and environmental pollution showed an inverted N-curve relationship, while financial efficiency has a U-shaped curve relationship with it.

### Literature review on the impact of digital finance on economic development

Although the impact of financial development on environmental pollution has been well discussed, it has generally focused on the development of the traditional financial sector, and few studies have addressed the impact of digital finance on carbon emissions reduction, which is the focus of this paper. It is worth noting that most of the studies on digital finance at this stage have focused on the relationship between digital finance and economic growth, which undoubtedly provides an important reference for the argument on how digital finance affects carbon emissions, given the close relationship between economic growth and carbon emissions. As a new type of service industry that integrates traditional finance with digital technology, digital finance can significantly expand the coverage of financial services and thus exert its inclusive effect, while relying on digital technology and the Internet platform can effectively improve the accuracy and efficiency of financial resource matching, enabling financial services to have both fairness and efficiency attributes, thus positively influencing high-quality development. Zhang et al. (4) found that digital financial development can promote inclusive economic growth by stimulating innovation and entrepreneurship, and the economic growth-enhancing effect of inclusive finance is more significant in rural areas.

Through a summary of the existing literature, the incremental effect of digital finance on high-quality economic development comes from three main sources: (1) The digital finance development helps to upgrade the industrial structure. Bai and Zhang (22) suggested that the inclusive nature of financial services can help optimize the allocation of primary capital, which in turn can promote the upgrading of industrial structure. Tang et al. (2) conducted an empirical analysis based on city-level panel data and find that the development of digital inclusive finance has a long-term and significant promotion effect on the upgrading of industrial structure. Second, the development of digital finance helps technological innovation. Li (23) suggested that the process of high-quality development is the transformation from factor-driven to innovation-driven, and innovation-driven cannot be effectively supported by the digital model of financial services. Teng and Ma (24) argued that digital financial development can enhance the innovation level of enterprises and thus provide new momentum for regional high-quality development. Tang et al. (2) find that digital financial development has a “structural” effect on enterprise. Xue and Hu (25) argued that digital finance, with its connotation of financial technology, can play a positive role in channeling capital and other factors and improve the financial industry's ability to serve the traditional economy. Li and Cheng (26) suggested that the application of digital technology has expanded the financing channels of enterprises and promoted capital accumulation and capital utilization efficiency through non-bank credit intermediation.

However, Huang (1) pointed out that there are many antecedents to the impact of digital finance on economic development, and that differences in both economic and institutional variables can lead to differential impacts of digital finance on high-quality development. Cao (27) considered that the integration capacity of China's industrial internet platform system is limited at this stage, and the degree of digitization of enterprises is generally low, thus limiting the development of enterprises and digital financial services which makes it difficult to promote industrial economic growth. Jiang and Sun (28) similarly found that the development of digital economy based on digital finance has a long-term crowding-out effect on the growth of the real economy, and the impact of digital finance on the real economy has become a non-negligible obstacle affecting the high-quality development of China's economy.

Through the above-mentioned literature, it is easy to find that there is a large research gap to examine the impact of digital finance in the environmental field, and no relevant literature has been found to examine the emission reduction effect of digital finance development in the context of the current “double carbon” targets of carbon peaking and carbon neutrality. In addition, current research often decomposes the impact of financial development on environmental pollution into scale and technology effects, but lacks the examination of specific transmission paths and the analysis of the dynamic change characteristics of the dominant paths. Based on this, this paper constructs a theoretical model of the impact of digital finance on manufacturing industry emission reduction, examines the emission reduction effect of digital finance on manufacturing industry from both theoretical analysis and empirical test.

### Mechanistic analysis and research hypotheses

Unlike the traditional financial services sector, the development of digital finance has both a “crowding out” effect and an “incentive effect” on the manufacturing sector, which is typical of the real economy. At the early stage of digital finance development, it tends to channel capital and other factors to the digital economy industry sector, while the traditional real economy such as manufacturing industry is vulnerable to the “crowding out” effect of digital economy industry development due to the generally low degree of digitalization and imperfect digital integration system. At this time, the development of digital finance has limited the technological product development and value creation of the manufacturing technology R&D sector, and the value of the parameter is on the negative side. However, along with the expansion of service capacity and scope, digital finance contributes to the deepening of the integration of the traditional real economy and the digital economy, thus creating an “incentive” impact on the economic growth of the manufacturing sector. The value of the sum parameter tends to change from negative to positive. It is thus inferred that the marginal impact of digital finance development on the economic growth of the manufacturing industry shows a dynamic pattern of “first inhibiting, then promoting.” In addition, considering that industry correlation can affect the marginal reduction effect of economic growth, it means that the marginal reduction effect of digital finance development may also be affected by industry correlation. Digital finance development may lead to spillover reduction between industries.

The U-shaped impact of digital finance on economic growth and the inverted U-shaped impact of economic growth on carbon intensity are analyzed together, and the paper suggests that the impact of digital finance on carbon intensity can be summarized in three stages: in the early stage, digital finance tends to crowd out the development of the real economy, such as manufacturing, and achieves carbon emission reduction through the “scale effect” of shrinkage; in the middle stage In the middle stage, digital finance can promote economic growth in the manufacturing sector by easing the financing constraint, which increases carbon intensity based on the “scale effect” of expansion; in the mature stage, the development of digital finance helps drive digital transformation and innovation and research and development of green technologies, driving carbon reduction through the “technology effect In the mature stage, the development of digital finance can help drive digital transformation and green technology innovation and research, and promote carbon reduction through the “technology effect.” Accordingly, the theoretical hypotheses of this paper can be formulated.

#### Hypothesis 1

Digital financial development has an inverted-N type of emission reduction effect on manufacturing.

#### Hypothesis 2

The emission reduction effect of digital finance development depends on the combined effect of the “technology effect” and the “scale effect,” and there are dynamic changes in the dominant effect in different time periods.

## Empirical research

### Benchmark regression model

Based on the above analysis of the mechanisms by which digital finance affects carbon emission reduction, this paper examines the impact of digital finance development on the carbon intensity of the manufacturing sector at the enterprise level, with the following benchmark model.


(1) $$\begin{array}{l} cc_{ijt}=\alpha_{0}+\alpha_{2}digital_{ijt}+\alpha_{3}digital_{ijt}^{2}+\alpha_{4}digital_{ijt}^{3}+\alpha_{5}X_{ijt}\\ \;+\delta_{r}\times time+\omega_{t}\times time+\gamma_{t}+\sigma_{i}+\varepsilon_{ijrt} \end{array}$$

where i, j, r and t denote listed manufacturing enterprises, industry, city and year respectively; denote the explanatory variable carbon intensity of manufacturing enterprises; and denote the digital finance development index. Considering the inverse N-shaped relationship between digital financial development and carbon intensity proposed in the hypothesis of this paper, this paper introduces both its quadratic and cubic terms into the benchmark model; denotes the random perturbation term, and is used to control for the time trend effect of region and time, respectively, while and denotes the time fixed effect and firm fixed effect, respectively. Meanwhile, other economic characteristics X that may affect the carbon intensity of manufacturing firms are controlled for in this paper, including firm age, firm nature and capital intensity.

### Variable description

#### The carbon emission intensity (cc)

As carbon emission data at the level of listed companies are difficult to obtain directly, this paper refers to Ju (29) and uses the energy consumption data disclosed in the social responsibility reports of listed companies to estimate their total carbon emissions. The resource consumption indicators involved include energy (converted to standard coal), gasoline, diesel, natural gas, piped gas, purchased electricity and purchased heat.

#### The digital financial development (digital)

This paper mainly adopts the Digital Inclusive Finance Development Index of Peking University's Digital Finance Research Center to characterize the development of digital finance, which measures the level of digital financial development in terms of breadth of coverage, depth of use and degree of digital support services. The index measures the level of digital financial development in terms of breadth of coverage, depth of use and the degree of digital support services, including electronic accounts such as internet payment accounts. The index is based on Ant Financial Services' big data on transaction accounts and is highly reliable (30).

#### The control variables

Referring to related studies on the productivity of listed manufacturing industries (31–40) this paper introduces a set of control variables: enterprise age (*AGE*) is represented by the difference between 2019 and the year of establishment of the enterprise; enterprise ownership (*ATTR*) is represented by a 0–1 dummy variable. One for state-owned enterprises and zero for non-state-owned enterprises; return on net assets (*ROE*) is represented by the ratio of after-tax profit to net assets of the company; gearing ratio (*LVE*) is expressed as the ratio of total liabilities to tangible assets; operating income growth rate (*REVE*) is expressed as the ratio of current operating income to previous operating income; equity concentration (*CR*) is expressed as the ratio of the largest shareholder's holding; and capital intensity (*CAID*) is expressed as the ratio of fixed assets to the number of employees in the firm.

### Data sources

A balanced panel dataset with a study period of 2011–2019 was established through city-firm level data matching. Among them, after eliminating samples with more missing values in the five-year period. Among them, various financial data related to enterprises were obtained from the wind database, enterprise patent data from the CNRDS database, enterprise green technology patent data from the national patent database, and energy consumption data mainly from listed companies' Corporate Social Responsibility Report, etc. In order to overcome the influence of outliers, this paper also carried out the tailing process for continuous variables below 1 and above 99% of the quantile.

## Analysis and discussion of empirical results

### Baseline regression analysis

The results in Table 1 show that the primary, secondary and tertiary terms of digital financial development pass the test at least at the 10% significance level with negative, positive and negative values, which means that digital financial development has a non-linear effect of “first suppressing, To analyze the possible reasons for this, it is difficult to form an effective interface between digital finance development and the real economy, such as the manufacturing industry, at the initial stage, and its financial services have an obvious bias toward the digital economy industry. Due to the relatively limited integration of the digital economy with the traditional economic development, the real economy tends to be “crowded out,” so the initial stage of digital financial development can promote the reduction of emissions in the manufacturing industry based on the “scale effect” of reduced investment, etc.; the medium-term stage of digital financial development The development of digital finance in the middle stage can alleviate the financing constraints of manufacturing enterprises based on its inclusive characteristics, and the integration of the digital economy with the real economy, such as manufacturing, further promotes the in-depth application of digital finance in the manufacturing sector, which will undoubtedly contribute to the expansion of the scale of investment in manufacturing enterprises and the increase in total energy consumption, thus hindering the reduction of emissions in the manufacturing sector based on the “scale effect” of expansion The development of digital finance in the mature stage can not only provide sufficient financial support for green innovation, but also force manufacturing enterprises to actively carry out digital transformation, in which the emission reduction effect of the “technology effect” exceeds that of the expansion of the “scale effect” on The carbon intensity of the expansion will ultimately contribute to the reduction of emissions in the manufacturing sector. Above results partly prove the Hypothesis 1, which states that the development of digital finance has an inverted N-curve relationship with the carbon intensity of manufacturing firms.

**Table 1:** Estimated results of the impact of digital finance on the carbon intensity of the manufacturing sector.

	**(1)**	**(2)**	**(3)**	**(4)**	**(5)**	**(6)**
*digital*	−0.2471^***^ (−3.53)	−0.2503^***^ (−3.04)	−0.0337^***^ (−2.85)	−0.0350^***^ (−3.01)	−0.0619^***^ (−4.14)	−0.0654^***^ (−4.09)
*digital* ^2^	−0.1206^*^ (−1.88)	−0.1254^*^ (−1.69)	0.0192^**^; (2.04)	0.0171^**^ (2.25)	0.0308^***^ (2.72)	0.0273^**^ (2.44)
*digital* ^3^	−0.0375 (−1.42)	0.0366 (1.34)	−0.0041^*^ (−1.75)	−0.0058^*^ (−1.68)	−0.0083^**^ (−1.96)	−0.0100^*^ (−1.78)
Control variables	NO	YES	YES	YES	YES	YES
Firm fixed effects	NO	NO	YES	YES	YES	YES
Year fixed effects	NO	NO	NO	YES	YES	YES
Province x time trend fixed effects	NO	NO	NO	NO	YES	YES
Industry x time–trend fixed effects	NO	NO	NO	NO	NO	YES
Adjusting for R^2^	0.4351	0.4647	0.4421	0.4972	0.5013	0.5126

***, ** and * denote tests passed at the 1, 5 and 10% significance levels.

### Robustness tests

In order to ensure the reliability of the analysis results, this paper uses a series of robustness tests to ensure the reliability of the estimation results, and the results are shown in Table 2. (1) Core explanatory variables are treated with a one-period lag. In order to overcome the potential endogeneity between digital financial development and carbon intensity of manufacturing firms, this paper uses digital financial development with a one-period lag as the core explanatory variable. The results show that the impact of digital financial development on the carbon intensity of manufacturing enterprises is still inverted-N. (2) Replacement of the explanatory variables. Carbon emission reduction includes both the reduction of carbon intensity and the reduction of total carbon emissions. Compared with the carbon intensity indicator, which portrays carbon emissions from the perspective of production efficiency, the total carbon emissions indicator can reflect the carbon emissions level of manufacturing enterprises from the perspective of production scale, so this paper chooses the total carbon emissions indicator as a new explanatory variable. The results show that the development of digital finance has an inverse N-shaped effect on the carbon intensity of manufacturing enterprises. This finding not only fully supports the emission reduction effect of digital finance development, but also reveals that digital finance development has the dual carbon emission reduction effect of “total emission reduction” and “intensity reduction.” (3) A sub-dimensional study based on the breadth of coverage, depth of use and degree of digital support services. Specifically, the impact of both the breadth of digital finance coverage and the depth of digital finance use on the carbon intensity of manufacturing enterprises remains consistent in an inverted-N pattern. In contrast, the impact of the extent of digital finance services on the carbon intensity of manufacturing enterprises is only monotonically pro-increasing, thus proving that the inverted-N impact of digital finance development on the carbon intensity of manufacturing enterprises mainly comes from the breadth of digital finance coverage and depth of use. It is easy to understand that the deepening of the application and promotion of digital finance has deepened the reliance of the manufacturing industry on digital finance, and its emission reduction effect has been highlighted, while the convenience of digital financial services mainly affects individual residents, and manufacturing enterprises are relatively weakly affected by it.

**Table 2:** Results of robustness analysis.

	**(1)**	**(2)**	**(3)**		
	**One period lag treatment**	**Replacement of explanatory variables**	**Covering the breadth dimension**	**Depth of use dimension**	**Level of service dimension**
*digital*	−0.0702^***^ (−3.98)	−0.0527^***^ (−3.26)	−0.0513^***^ (−2.62)	−0.0872^***^ (−4.49)	0.0252^*^ (1.69)
*digital* ^2^	0.0266^**^ (2.15)	0.0209^***^ (2.96)	0.0216^*^ (1.89)	0.0302^**^ (2.38)	0.0129 (1.30)
*digital* ^3^	−0.0092^*^ (−1.85)	−0.0018^*^ (−1.82)	−0.0047^*^ (−1.70)	−0.0081^**^ (−1.97)	−0.0026 (−0.85)
Control variables	YES	YES	YES	YES	YES
Firm fixed effects	YES	YES	YES	YES	YES
Year fixed effects	YES	YES	YES	YES	YES
Province x time trend fixed effects	YES	YES	YES	YES	YES
Industry x time–trend fixed effects	YES	YES	YES	YES	YES
Adjusting for R^2^	0.5798	0.7622	0.6214	0.5883	0.3418

***, ** and * indicate passing the test at the 1, 5 and 10% significance levels.

There is a possible two-way causal relationship between the development of digital finance and the carbon intensity of the manufacturing sector. On the one hand, as an important support for economic growth, digital finance development can improve the supply of funds and promote the expansion of production capacity by alleviating financing constraints, which in turn has an impact on the carbon emissions of the manufacturing sector; on the other hand, the manufacturing sector needs to base its own emission control and emission reduction on clean technology research and development, and the demand for funds for technology research and development The demand for funds for technological research and development will undoubtedly force manufacturing enterprises to accelerate their digital transformation and deepen the application of digital finance. Although the previous analysis overcomes the potential endogeneity problem to a certain extent, the variables selected are not specific instrumental variables for digital finance development, so this paper selects specific instrumental variables for endogeneity analysis based on physical geography and historical data. Considering the inverse N-shaped relationship between digital finance development and carbon intensity of manufacturing firms, three instrumental variables are introduced in this paper. (1) The number of post offices per million people (POST) and the number of fixed-line telephones per million people (TEL) in 1984. The history of digital finance is also the history of the development of the digital technology on which it is based, with the Internet and other digital technologies as its core, thus enabling the development of online financial services and the expansion of the scope of services. In fact, the essence of digital technology is the change in the way information is transmitted. In the early days, information transmission was mainly achieved through post offices and fixed telephones, so areas with a dense distribution of post offices and a large number of fixed telephones tended to have more developed information communication, and the rise of digital technology and the development of digital finance was more likely to occur in these places. Considering that data on the number of post offices and fixed telephone ownership at the city level in China can be traced back as far as 1984 in the China Urban Statistical Yearbook, this paper chooses the number of post offices per million people (POST) and fixed telephones per million people (TEL) in 1984 as the specific instrumental variables. This historical data can effectively portray the level of regional communication development and meet the requirement of correlation, while at the same time it is difficult to influence the carbon intensity of the manufacturing industry at this stage due to the long period of time, and satisfies the assumption of exclusivity. (2) The spherical distance from each city to Hangzhou (DIST). The development of digital finance, represented by Alipay, originated in Hangzhou, which is therefore in a leading position, and it can be expected that the closer in geographic distance to Hangzhou, the better the degree of digital finance development should be. At the same time, geographical distance as a typical physical geographical feature is not closely related to economic and social factors, thus satisfying the correlation and exclusivity assumptions as well. In this paper, the spherical distance from each city to Hangzhou is calculated using a Geographic Information System (GIS). To apply the above instrumental variables to the balanced panel data, the interaction terms of the number of post offices/million people in 1984, the number of landline phones/million people and the spherical distance from each city to Hangzhou are constructed in turn as time-series instrumental variables with the digital financial inclusion index.

Table 3 reports the estimation results of the endogeneity analysis. From the first stage regression results, the number of post offices per million people and the number of landline telephones have a significant positive correlation with digital financial development overall, while the spherical distance from each city to Hangzhou has a significant negative correlation with digital financial development, thus demonstrating that all three instrumental variables selected in this paper are closely related to digital financial development. The results of the second stage of estimation show that there is an inverse N-shaped effect between digital financial development and carbon intensity of manufacturing enterprises, which implies that the effect of digital financial development on carbon intensity of manufacturing enterprises remains robust after overcoming the endogeneity problem.

**Table 3:** Results of the endogeneity analysis.

	**Stage 1**	**Stage 1**	**Stage 1**	**Stage 2**
*digital*				−0.0128^**^ (−2.15)
*digital* ^2^				0.0076^*^ (1.88)
*digital* ^3^				−0.0008^*^ (−1.74)
*TEL*	0.0165^*^ (1.78)	0.0112 (1.59)	0.0205^*^ (1.70)	
*POST*	0.0202^**^ (2.08)	0.0246^**^ (1.94)	0.0175^**^ (2.23)	
*DIST*	−0.0385^***^ (−2.94)	−0.0186^***^ (−2.72)	−0.0547^***^ (−3.13)	
Control variables	YES	YES	YES	YES
Firm fixed effects	YES	YES	YES	YES
Year fixed effects	YES	YES	YES	YES
Province x time trend fixed effects	NO	NO	NO	NO
Industry x time trend fixed effects	YES	YES	YES	YES

***, **, * indicate passing the test at the 1, 5 and 10% significance levels respectively. This paper does not control for area fixed effects given the difficulty of varying geographical distance over time for the geographic data instrument variable.

### Heterogeneity analysis

Considering the differences in regional development and enterprises' own attributes, this paper further examines the heterogeneous characteristics of the emission reduction effect of digital finance at the enterprise level and regional level, and the results are shown in Table 4. At the enterprise level, this paper examines the differential emission reduction effects of digital finance development in the enterprise age dimension, the enterprise ownership dimension and the enterprise scale dimension in turn. (1) Firm age dimension. The impact of digital finance development on the carbon intensity of young enterprises is inverted N-shaped, while the impact on the carbon intensity of mature enterprises is U-shaped, indicating that it is difficult for digital finance development to form a long-term emission reduction effect in mature enterprises at this stage. At the same time, comparing the intensity of the impact of digital finance development on both, we can see that the carbon intensity of young enterprises is more deeply affected by digital finance development. The reason for this is that compared to mature enterprises, young enterprises are more receptive to digital financial services and other Internet technology applications, and tend to be more open and forward thinking in their management, while mature enterprises are relatively conservative in their business models and the application of digital financial services within them has lagged. (2) Enterprise ownership dimension. Unlike non-state-owned enterprises, whose carbon intensity is affected by the development of digital finance in an inverted N-shape, the carbon intensity of state-owned enterprises is correlated with the development of digital finance in an inverted U-shape. The carbon intensity of digital finance increases at the early stage of development, while it decreases when the development of digital finance reaches a mature stage. The possible reason for this phenomenon is that SOEs have strong financing guarantees and policy support, and it is difficult to squeeze out manufacturing SOEs from the financing constraints caused by the initial development of digital finance. Thus, compared to non-SOEs, SOEs can move more quickly into the digital emissions reduction phase by actively deepening their use of digital financial services. (3) Enterprise size dimension. The inverse effect of digital finance development on emission reduction in the manufacturing industry is concentrated in large and medium-sized enterprises, while in small-sized enterprises, it is only an increase in carbon intensity. This paper suggests that small-scale enterprises are generally in the initial profit-seeking stage, with weak financing capacity and insufficient emphasis on technological research and development, and therefore the financial factors obtained by small-scale enterprises through digital finance are more inclined to capacity expansion.

**Table 4:** Analysis of heterogeneity.

	**Young enterprises**	**Mature enterprises**	**State-owned enterprises**	**Non-State-owned enterprises**	**Small-scale enterprises**	**Large and medium-sized enterprises**
*digital*	−0.0758^***^ (−4.44)	−0.0545^**^ (−2.16)	0.0492^***^ (2.72)	−0.0518^***^ (−5.28)	0.0184^***^ (4.09)	−0.0622^***^ (−3.99)
*digital* ^2^	0.0311^***^ (2.87)	0.0115^*^ (1.87)	−0.0170^**^ (−2.44)	0.0263^***^ (2.79)	0.0066 (1.09)	0.0283^***^ (2.77)
*digital* ^3^	−0.0093^**^ (−1.95)	−0.0007 (−1.26)	−0.0076 (−1.23)	−0.0055^**^ (−2.00)	−0.0008 (−0.85)	−0.0061^**^ (−1.98)

***, ** and * indicate passing the test at the 1, 5 and 10% significance levels respectively.

### Mechanism analysis

In order to examine the intrinsic mechanism of digital financial development affecting carbon emissions, this paper constructs the following panel threshold model (PTM) using digital financial development as the threshold variable to empirically examine the phasing of digital financial development affecting carbon intensity of manufacturing enterprises.


(2) $$\begin{array}{l} cc_{ijt}=\;\gamma_{0}+\gamma_{1}digital_{ijt}\times \;I(digital_{ijt}\leq \lambda )+\;\gamma_{2}digital_{ijt}\;\\ \;\times \;I(digital_{ijt}>\lambda )+\gamma_{3}X_{ijt}+\delta_{r}\times time+\omega_{r}\times time+\gamma_{t}\\ \;+\;\sigma_{i}+\varepsilon_{ijrt} \end{array}$$

where I(·) denotes the indicator function, I(·) = 1 when the function holds and 0 otherwise; and λ denotes the threshold value. The threshold effect model sets the original hypothesis as H0: γ2 = γ3, which means that the original hypothesis is accepted and there is no threshold effect; rejection of the original hypothesis indicates that there is a threshold effect. After passing the single threshold model test, the double threshold model test is continued, and if the double threshold model is significant then the triple threshold model test is carried out by analogy, otherwise the model is a single threshold model. In this paper, the specific F-statistic values of the above equation are estimated under 300 bootstrap self-sampling to determine whether the threshold effect model exists, as well as the number and value of the thresholds, and the results are reported in Table 5. Digital financial development can pass the double threshold test. The impact of digital financial development on the carbon intensity of manufacturing enterprises can be classified into three stages: ‘primary', ‘intermediate' and ‘mature'. The development of digital finance can be classified into three stages: ‘primary', ‘intermediate' and ‘mature'. Meanwhile, in the primary and mature stages, the impact of digital finance development on the carbon intensity of manufacturing enterprises is significantly negative at – 0.0351 and – 0.0102 respectively, while in the intermediate stage, the impact is significantly positive (0.0194), which not only fully supports the conclusion that digital finance development has a “suppressing, then increasing, then suppressing” effect on the carbon intensity of manufacturing enterprises. This finding not only confirms that digital finance development has an inverted N-shaped impact on the carbon intensity of manufacturing enterprises, but also indicates that the impact of digital finance development on the carbon intensity of manufacturing enterprises can be divided into three stages (Table 6).

**Table 5:** Threshold effect test and threshold estimation results.

	**Threshold**	**F–statistic**	**10%**	**5%**	**1%**
**Part I**					
Single threshold	129.54	43.86 [0.02]	23.21	30.01	50.10
Double threshold	280.42	19.52 [0.01]	22.03	25.42	33.25
Triple threshold	340.71	11.67 [0.76]	30.75	35.18	45.19
**Part II**					
*digital* < λ_1_	−0.0351^**^(−1.98)				
λ_1_<*digital* < λ_2_	0.0194^**^(2.96)				
*digital*>λ_2_	−0.0102^*^(−1.85)				

***, ** and * indicate passing the test at the 1, 5 and 10% significance levels respectively.

**Table 6:** Results of the analysis of intrinsic mechanisms.

	**Primary stage**			**Intermediate stage**			**Mature stage**		
	**(*****digital*****<**λ****_**1**_ **)**			**(**λ****_**1**_ **<*****digital*****<**λ****_**2**_ **)**			**(*****digital*****>**λ****_**2**_ **)**		
	***invest***	***gtech***	***cc***	***invest***	***gtech***	***cc***	***invest***	***gtech***	***cc***
*digital* × *KZ*	−0.5264^*^ (−1.91)	−0.0204 (−0.44)		1.2033^***^ (2.88)	0.0417^*^ (1.90)		0.8402^*^ (1.86)	0.0197^***^ (2.66)	
*digital* × *ie*		0.0288 (0.44)			0.0417 (1.50)			0.0202^*^ (1.86)	
*digital* × *KZ* × *invest*			−0.0149^*^ (−1.75)			0.0228^***^ (3.15)			0.0127^**^ (2.20)
*digital* × *KZ* × *gtech*			−0.0112 (−0.85)			−0.0076^*^ (−1.70)			−0.0136^**^ (−1.98)
*digital* × *ie* × *gtech*			0.0073 (0.89)			−0.0057 (−1.53)			−0.0081^**^ (−2.24)
Control variables	YES	YES	YES	YES	YES	YES	YES	YES	YES
Firm fixed effects	YES	YES	YES	YES	YES	YES	YES	YES	YES
Year fixed effects	YES	YES	YES	YES	YES	YES	YES	YES	YES
Province x time trend fixed effects	YES	YES	YES	YES	YES	YES	YES	YES	YES
Industry x time trend fixed effects	YES	YES	YES	YES	YES	YES	YES	YES	YES

***, ** and * denote passing the test at the 1, 5% and 10% levels of significance respectively.

In the early stages, the development of digital finance can reinforce the financing constraints of manufacturing enterprises and lead to a reduction in their investment scale, and has a potential inhibiting effect on corporate green innovation. This is mainly due to the fact that the financial services provided by digital finance in the early stages of development are more inclined to the digital economy, which is driven by digital technology and digital finance, and the development of the digital economy can create a crowding-out effect on the real economy, such as the manufacturing sector. Moreover, although digital finance can fuel green innovation by forcing the digital transformation of enterprises, this pro-increase effect is not significant in the primary stage. On this basis, only the interaction between digital finance, financing constraints and investment scale (−0.0149) has a significant negative impact on the carbon intensity of manufacturing firms in the primary stage, so the reduced ‘scale effect' in the primary stage is the key to the reduction in carbon intensity of manufacturing firms due to the development of digital finance.

In the intermediate stage, thanks to the deepening of the application of digital finance and the effective play of the inclusive feature, digital finance can effectively alleviate the financing constraints of manufacturing enterprises, thus providing sufficient financial support for the expansion of investment scale and enterprise technology research and development. The impact of digital finance development on both investment scale and green innovation is significantly positive, but the effect on investment scale is much stronger than that on green innovation. At the same time, the effect of digital finance development on green innovation based on the digital transformation of enterprises is still not significant, which may be related to the longer period of technology research and development, application and diffusion. At this point, the effect of the interaction term (*digital* × *KZ* × *invest*) between digital finance, financing constraints and investment scale on the carbon intensity of manufacturing enterprises is significantly positive (0.0228), while the effect of the interaction term (*digital* × *KZ* × *gtech*)between digital finance, financing constraints and investment scale is significantly negative (−0.0076), which indicates that digital finance development in the intermediate stage can induce the “scale effect” and “technology effect” of expansion This indicates that digital finance development in the intermediate stage can induce the “scale effect” and the “technology effect” to jointly affect the carbon intensity of manufacturing enterprises, but since the carbon intensity increase due to the scale effect is significantly greater than the carbon intensity decrease due to the technology effect, digital finance development in the intermediate stage eventually manifests itself as a carbon intensity-enhancing effect.

In the mature stage, digital finance development can still effectively alleviate the financing constraint and expand the scale of corporate investment, but the degree of enhancement decreases compared to the intermediate stage. At the same time, the development of digital finance can also significantly increase the level of green innovation by alleviating the financing constraint and forcing the digital transformation of enterprises. In this stage, digital finance development can also induce the “scale effect” of expansion to increase the carbon intensity of manufacturing enterprises, but the “technology effect” driven by the easing of financing constraints and digital transformation can significantly reduce the carbon intensity of manufacturing enterprises, and the sum of The “technology effect” is significantly stronger than the “scale effect,” which ultimately results in a positive carbon reduction effect for manufacturing enterprises.

## Conclusions and recommendations

This paper links carbon emissions and digital finance variables. This paper introduces carbon emissions and digital finance variables into the endogenous growth Romer model that includes knowledge output, and theoretically analyzes the inverted-N relationship between digital finance development and carbon intensity; then, by matching city-manufacturing supervisor firm data, we empirically test the impact of digital finance development on the carbon intensity of manufacturing enterprises, and based on the “scale effect” and “scale effect,” we also examine the impact of digital finance development on the carbon intensity of manufacturing enterprises. The main conclusions are as follows: The impact of digital finance on the carbon intensity of manufacturing enterprises is based on the “scale effect” and “technology effect.” The main findings are as follows:

1. There is a significant inverse-N relationship between digital finance development and carbon intensity of manufacturing enterprises. The dynamic impact on carbon intensity of manufacturing industries is “first inhibited, then promoted, then inhibited.”
2. There is typical heterogeneity in the impact of digital financial development on the carbon intensity of the manufacturing sector. On the firm dimension, the emission reduction effect of digital financial development on young manufacturing firms is better than that of mature firms in the long run. Similarly, the emission reduction effect of digital financial development on large and medium-sized enterprises is more prominent compared to small-sized enterprises. State-owned enterprises (SOEs), with their policy support, are better able to benefit from the financing dividend brought by digital finance development, thus showing an inverted U-shaped emission reduction effect, while non-SOEs show an inverted N-shaped emission reduction effect.
3. The inverse N-shaped impact of digital finance development on manufacturing carbon intensity depends on the combined effect of “scale effect” and “technology effect.” In the primary stage, the development of digital finance will aggravate the financing constraints faced by manufacturing enterprises, thus forming a “crowding-out effect” on the development of the manufacturing sector, and this reduced “scale effect” will help reduce carbon emissions; in the intermediate stage, the development of digital finance can On the other hand, it can provide sufficient financial support for technology research and development and force the digital transformation of enterprises, however, due to the long cycle of technology research and development, the “technology However, due to the long cycle of technology development, the “technology effect” is much smaller than the “scale effect” of expansion, so digital finance development can enhance the carbon intensity of the manufacturing industry; although digital finance development in the mature stage can also induce the “scale effect” of expansion, the “technology effect” caused by digital finance development is much smaller than the “scale effect” of expansion. Although digital finance development at a mature stage can also induce the “scale effect” of expansion, the “technology effect” caused by digital finance development is significantly enhanced and exceeds the “scale effect” of expansion.

Based on the above research findings, this paper mainly puts forward the following policy recommendations: The government departments should actively guide enterprises to develop an effective interface with digital financial services, and enterprises themselves should actively seek the optimal path for digital transformation, so as to promote the development of digital finance in the manufacturing sector to break through the “emission reduction inflection point” as soon as possible and build a low-carbon model for long-term emission reduction in the manufacturing sector. Specifically, the development of digital finance needs to clarify the three basic principles of “inclusiveness,” “low carbon” and “synergy.”

Firstly, digital financial services should avoid an excessive bias toward the digital economy, weaken as much as possible the crowding-out effect of the digital economy on the real economy, and strengthen the deep integration of the digital economy and the real economy. Secondly, digital financial development should be combined with the current development visions of “carbon peaking” and “carbon neutrality,” and strengthen the development of digital finance. Finally, the formulation of digital finance development strategies should vary from time to time, from place to place and from enterprise to enterprise. In view of the differential impact of digital finance development on the carbon intensity of the manufacturing industry at the primary, secondary and mature stages, digital finance should develop differentiated development strategies. In order to fully unleash the emission reduction effect of digital finance in the manufacturing sector, it is necessary to strengthen the financial support of digital finance for the research and development of green technology innovation on the one hand, and accelerate the digital transformation of manufacturing enterprises on the other hand, so as to achieve long-term emission reduction by enhancing the effect of technology.

## Research outlook

The research deficiencies and improvement plans are mainly as follows: First, this paper only focuses on the impact of digital finance on the manufacturing sector, and future research will be expanded to the entire industrial field. Second, the role of digital finance in affecting the carbon intensity of the manufacturing industry is not clear enough. Therefore, the mechanism analysis needs to be in-depth. Third, the development of digital finance can lead to significant industry spillover effects, while this study ignores this point. The spillover effect between industries needs to be further taken into account in future study.

## Data availability statement

The data is available in the wind database, the CNRDS database, the national patent database and the listed companies' Corporate Social Responsibility Report, etc.

## Ethics statement

This study was reviewed and approved by the College of Biological and Agricultural Engineering, Jilin University.

## Author contributions

YY designed the research, conducted the research, analyzed the data, and wrote the paper.

## Conflict of interest

The author declares that the research was conducted in the absence of any commercial or financial relationships that could be construed as a potential conflict of interest.

## Publisher's note

All claims expressed in this article are solely those of the authors and do not necessarily represent those of their affiliated organizations, or those of the publisher, the editors and the reviewers. Any product that may be evaluated in this article, or claim that may be made by its manufacturer, is not guaranteed or endorsed by the publisher.
